# *In vitro* Optimization of Ceftazidime/Avibactam for KPC-Producing *Klebsiella pneumoniae*

**DOI:** 10.3389/fmicb.2021.618087

**Published:** 2021-03-04

**Authors:** Yanqin Huang, Tiffany Wu, Omar Perez, Amisha P. Rana, Liang Chen, Barry N. Kreiswirth, Michael J. Satlin, Zackery P. Bulman

**Affiliations:** ^1^Department of Pharmacy Practice, College of Pharmacy, University of Illinois at Chicago, Chicago, IL, United States; ^2^Center for Discover and Innovation, Hackensack Meridian Health, Nutley, NJ, United States; ^3^Department of Medical Sciences, Hackensack Meridian School of Medicine, Nutley, NJ, United States; ^4^Department of Medicine, Weill Cornell Medicine, New York, NY, United States

**Keywords:** ceftazidime/avibactam, *Klebsiella pneumoniae*, carbapenemase, synergy, time kill assay and antibacterial activity

## Abstract

Ceftazidime/avibactam is an important treatment option for infections caused by *Klebsiella pneumoniae* carbapenemase-producing *K. pneumoniae* (KPC-Kp), however, resistance can emerge during treatment. The objective of the study was to define the ceftazidime/avibactam concentrations required to suppress bacterial regrowth in ceftazidime/avibactam susceptible isolates and identify active therapies against ceftazidime/avibactam-resistant KPC-Kp. Time-kill assays were performed against twelve ST258 KPC-Kp isolates that harbored *bla*_KPC__–__2_ or *bla*_KPC__–__3_. Nine KPC-Kp isolates (KPC-Kp 5A, 6A, 7A, 8A, 9A, 24A, 25A, 26A, and 27A) were susceptible to ceftazidime/avibactam, two (KPC-Kp 6B and 7B) were ceftazidime/avibactam resistant and meropenem susceptible, and one (KPC-Kp 1244) was resistant to both ceftazidime/avibactam and meropenem. Sequencing of the *bla*_KPC_ genes revealed mutations in KPC-Kp 6B (D179Y substitution) and 7B (novel 21 base pair deletion) that both affected the Ω-loop encoding portion of the gene. Time-kill assays showed that against ceftazidime/avibactam-susceptible KPC-Kp, ceftazidime/avibactam concentrations ≥40/7.5 mg/L caused mean 5.42 log_1__0_CFU/mL killing and suppressed regrowth. However, regrowth occurred for some KPC-Kp isolates with a ceftazidime/avibactam concentration of 20/3.75 mg/L. Against ceftazidime/avibactam-resistant and meropenem-susceptible KPC-Kp 6B and 7B, bactericidal activity and synergy was observed for ceftazidime/avibactam in combination with meropenem ≤3.125 mg/L, while meropenem concentrations ≥50 mg/L were bactericidal as monotherapy. In contrast, clinically achievable concentrations of ceftazidime/avibactam were bactericidal against KPC-Kp 1244, which was ceftazidime/avibactam-resistant and meropenem-resistant due to outer membrane porin mutations and elevated *bla*_KPC_ expression. Achieving high ceftazidime/avibactam concentrations may help to suppress bacterial regrowth in the presence of ceftazidime/avibactam. The optimal treatment approach for ceftazidime/avibactam-resistant KPC-Kp likely depends on the mechanism of resistance. Additional studies are warranted to confirm these findings.

## Introduction

Carbapenem-resistant *Enterobacterales* (CRE) is associated with high mortality rates in patients with serious infections and remain a public health concern worldwide. Carbapenem resistance in *Enterobacterales* is most commonly conferred by carbapenemases, of which the *K. pneumoniae* carbapenemase (KPC) is the most common in the United States. β-Lactam/β-lactamase inhibitors with activity against KPC-Kp, such as ceftazidime-avibactam (CAZ/AVI), have been shown to improve clinical outcomes for patients with complicated urinary tract infections and intra-abdominal infections caused by KPC-Kp compared to older treatment options such as the polymyxins (van Duin et al., 2018). Although frequency of mutations conferring CAZ/AVI resistance *in vitro* remains low (10^–7^–10^–9^) (Livermore et al., 2018), resistance has already been observed during treatment (Shields et al., 2017a, 2018a).

Two primary mechanisms are responsible for CAZ/AVI resistance in KPC-Kp that do not also harbor metallo-β-lactamase (MBL) encoding genes. First, CAZ/AVI resistance can be caused by non-synonymous mutations in the *bla*_KPC_ gene that lead to amino acid substitutions within the KPC Ω-loop (Barnes et al., 2017; Shields et al., 2017a). The KPC Ω-loop encompasses amino acid positions 164 through 179 and some amino acid substitutions within this region enhance the KPC enzyme affinity for ceftazidime, which may prevent the binding of avibactam (Barnes et al., 2017). Interestingly, some of these mutations in the *bla*_KPC_ Ω-loop that cause CAZ/AVI resistance also restore carbapenem susceptibility (Shields et al., 2017a). A second cause of CAZ/AVI resistance in KPC-Kp is through the increased expression of *bla*_KPC_ in combination with diminished bacterial permeability to β-lactams (i.e., mutated or non-functional outer membrane porin channels) (Nelson et al., 2017). Increased gene copy number has been shown to drive increased *bla*_KPC_ expression while mutations in both *ompK35* and *ompK36* are considered important for reduced susceptibility to CAZ/AVI (Nelson et al., 2017). Optimal antimicrobial therapy for KPC-Kp isolates that are resistant to CAZ/AVI has not been defined and may depend on the mechanism of CAZ/AVI resistance. The objective of this study was to identify active antimicrobial therapies and optimize their concentrations to maximize bacterial killing and minimize bacterial regrowth of CAZ/AVI-susceptible and resistant KPC-Kp isolates.

## Materials and Methods

### Antibiotic Susceptibility and Molecular Mechanisms of Resistance

Twelve KPC-Kp isolates were used; nine were CAZ/AVI-sensitive (KPC-Kp 5A, 6A, 7A, 8A, 9A, 24A, 25A, 26A, and 27A) and three were CAZ/AVI-resistant (KPC-Kp 6B, KPC-Kp 7B, and KP1244) (Table 1). The susceptible KPC-Kp isolates were obtained from patients with hematologic malignancies who developed bacteremia and septic shock. KPC-Kp 6B and 7B were generated by serially passaging isolates KPC-Kp 6A and 7A, respectively, in Cation-Adjusted Mueller Hinton II Broth (CAMHB) with AVI (4 mg/L) and concentrations of CAZ that doubled every 24 h (starting at 0.5 mg/L and ending at 128 mg/L), as previously described (Livermore et al., 2015). MICs for each isolate were determined in triplicate with broth microdilution according to CLSI guidelines. CAZ/AVI-susceptible isolates at an inocula of ∼10^6^ CFU/mL were also plated on CAZ/AVI embedded agar plates (16/4 mg/L) to detect the presence of CAZ/AVI-resistant subpopulations at baseline.

**TABLE 1:** MICs, β-lactamases, and KPC amino acid changes for the tested KPC-Kp isolates.

Isolate	MIC, mg/L				β-lactamases	KPC amino acid changes
	CAZ	CAZ/AVI	IMI	MERO		
5A	>64 (R)	0.5/4 (S)	32 (R)	32 (R)	KPC-2, SHV-12, TEM-1	None
6A	>64 (R)	0.5/4 (S)	16 (R)	32 (R)	KPC-3, SHV-11	None
7A	>64 (R)	0.5/4 (S)	16 (R)	32 (R)	KPC-3, SHV-11, TEM-1	None
8A	>64 (R)	1/4 (S)	16 (R)	32 (R)	KPC-3, SHV-11, TEM-1	None
9A	32 (R)	0.125/4 (S)	8 (R)	16 (R)	KPC-2, SHV-11, TEM-1	None
24A	64 (R)	1/4 (S)	64 (R)	64 (R)	KPC-2	None
25A	64 (R)	0.5/4 (S)	16 (R)	32 (R)	KPC-3, TEM-1	None
26A	>64 (R)	1/4 (S)	>64 (R)	>128 (R)	KPC-2, SHV-11, TEM-1	None
27A	64 (R)	0.5/4 (S)	16 (R)	32 (R)	KPC-2, SHV-11, TEM-1	None
6B	>512 (R)	512/4 (R)	0.5 (S)	0.0625 (S)	KPC-3, SHV-11	D179Y in the KPC-3
7B	>512 (R)	512/4 (R)	0.25 (S)	0.0625 (S)	KPC-3, SHV-11, TEM-1	D163_L169del and N268_K269insKDD
1,244	>64 (R)	16/4 (R)	64 (R)	256 (R)	KPC-3, SHV-11, SHV-12	None*

R, resistant; S, susceptible; CAZ/AVI, ceftazidime/avibactam; IMI, imipenem; MERO, meropenem.* Increased bla_*KPC*__–__3_ expression, OmpK35/36 deficiency, and increased expression of the acrAB efflux pump.

PCR and Sanger sequencing with previously published primers were performed for detection of *bla*_*SHV*_, *bla*_TEM_, *bla*_CTX__–__M_, *bla*_NDM_, *bla*_VIM_, *bla*_IMP_, and *bla*_OXA__–__48_ genes (Poirel et al., 2011). A multiplex molecular-beacon real-time PCR assay was used to detect the presence and type of *bla*_KPC_ gene (Chen et al., 2011). Multilocus sequence typing (MLST) was performed as previously described (Diancourt et al., 2005). Resulting CAZ-AVI-resistant isolates KPC-Kp 6B and 7B were subjected to PCR and Sanger sequencing to determine the sequence of each isolate’s *bla*_KPC__–__3_ gene (forward primer 5′-ATGTCACTGTATCGCCGTCT-3′; reverse primer 5′-TTTTCAGAGCCTTACTGCCC-3′). KP1244 was previously determined to be resistant to CAZ/AVI through a combination of increased *bla*_KPC__–__3_ expression, OmpK35/36 deficiency, and increased expression of the *acrAB* efflux pump (Nelson et al., 2017).

Modified Hodge Tests (MHT) were performed to detect carbapenemase activity according to CLSI guidelines (CLSI, 2019) and as previously described (Cohen Stuart et al., 2010). MHT positive *K. pneumoniae* ATCC 1705 was used as a quality control strain. The CAZ-AVI-resistant isolates (KPC-Kp 6B and 7B), their parent isolates (KPC-Kp 6A and 7A), and *K. pneumoniae* ATCC 1705 were streaked onto a lawn of *E. coli* ATCC 25922 with a 10 μg meropenem disk. The plates were then incubated for 16–24 h at 37°C. An additional MHT was also performed with one adjustment to confirm the presence of non-carbapenemase β-lactamase activity in KPC-Kp 6B and 7B; a 30 μg ceftazidime disk replaced the meropenem disk. A positive MHT result was defined as a clover leaf-like indentation of the *E. coli* ATCC 25922 growing along the test organism streak within the disk’s zone of inhibition. A negative MHT result showed no growth of the *E. coli* 25922 along the test organism growth streak within the disk’s zone of inhibition. The tests were performed in triplicate.

### Time-Kill Assays

Time-kill assays were performed against each KPC-Kp isolate in CAMHB at a target starting inocula of ∼10^6^ CFU/mL, as previously described (Bulman et al., 2017). Viable colony counts were performed by obtaining samples after 0, 1, 2, 4, 6, 8, and 24 h of antibiotic exposure. A range of antibiotic concentrations above and below the *f*C_max_ were used against each isolate. To determine the CAZ/AVI concentration required to suppress regrowth of CAZ/AVI-susceptible KPC-Kp isolates, CAZ/AVI concentrations of 20/3.75, 40/7.5, 80/15 (*f*C_max_), and 160/30 mg/L were utilized. A 4:1 ratio of CAZ/AVI was selected to mimic the approximate concentration ratio observed in patients and based on the ratio of the drugs in the commercially available formulation (Li et al., 2019). To determine if CAZ/AVI acts synergistically with meropenem (MERO) for CAZ/AVI-resistant KPC-Kp isolates with mutations in the *bla*_KPC_ Ω-loop (KPC-Kp 6B and 7B), MERO concentrations of 0.78, 3.125, 12.5, 50 (*f*C_max_), and 200 mg/L were tested alone and in combination with CAZ/AVI at 80/15 mg/L (*f*C_max_). Lastly, to determine if increasing concentrations of CAZ/AVI can overcome CAZ/AVI resistance caused by increased *bla*_KPC_ expression, and porin deficiency (KP1244), CAZ/AVI concentrations of 16/2, 16/4, 16/8, 16/16, 8/4, 32/4, and 64/4 mg/L were utilized. Each experiment was performed at least two times and data are presented as the mean. Synergy was defined as a ≥2 log_10_ CFU/mL decrease by the combination compared to the most active agent alone. Bactericidal activity was defined as a ≥3 log_10_ CFU/mL reduction in viable bacterial count at 24 h compared with the initial inoculum.

## Results

### Antibiotic Susceptibility, Molecular Mechanisms of Resistance, and Modified Hodge Test

All *K. pneumoniae* isolates used in this study belonged to ST258. The nine CAZ/AVI-susceptible KPC-Kp isolates had CAZ MICs ≥32 mg/L,CAZ/AVI MICs ranging from 0.125/4 to 1/4 mg/L, MERO MICs ≥16 mg/L, and imipenem MICs ≥8 mg/L (Table 1). Five KPC-Kp isolates harbored *bla*_KPC__–__2_ whereas four harbored *bla*_KPC__–__3_. None of these isolates had CAZ/AVI-resistant subpopulations at baseline. Serial passaging KPC-Kp 6A (CAZ/AVI MIC = 0.5/4 mg/L) with increasing CAZ/AVI concentrations yielded CAZ/AVI-resistant isolate KPC-Kp 6B (CAZ/AVI MIC = 512/4 mg/L), which contained an D179Y substitution in the KPC-3 enzyme. Exposure of KPC-Kp 7A (CAZ/AVI MIC = 0.5/4 mg/L) to increasing CAZ/AVI concentrations resulted in KPC-Kp 7B (CAZ/AVI MIC = 512/4 mg/L), which contained a 21 base pair deletion and a 9 base pair insertion in the *bla*_KPC__–__3_ gene (yielding amino acid changes: D163_L169del and N268_K269insKDD). The nucleotide deletion resulted in deletion of the amino acids in positions 163–169, which are primarily in the KPC Ω-loop. Both KPC-Kp 6B and 7B became MERO-susceptible following these serial passaging experiments (MERO MICs = 0.0625 mg/L). In agreement with the MIC results, KPC-Kp 6B and 7B were MHT negative while their parent strains (KPC-Kp 6A and 7A) were MHT positive. The MHT experiment with a ceftazidime disk showed that KPC-Kp 6B and 7B harbored enzymes that displayed increased hydrolysis of ceftazidime (MHT positive) compared to their parent strains, which were both ceftazidime-MHT negative.

### Time-Kill Assays

**FIGURE 1:**
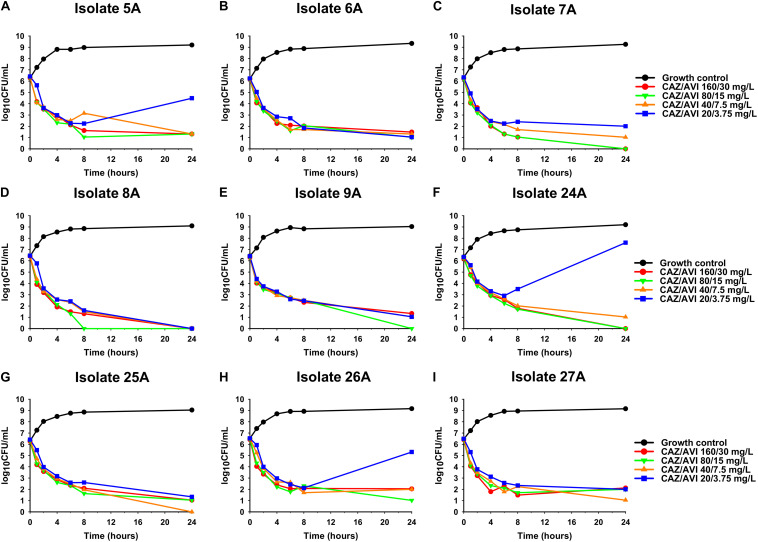
Time-kill assays over 24 h evaluating CAZ/AVI at concentrations of 20/3.75, 40/7.5, 80/15, and 160/30 mg/L against 9 clinical CAZ/AVI-susceptible KPC-Kp isolates **(A–I)**.

**FIGURE 2:**
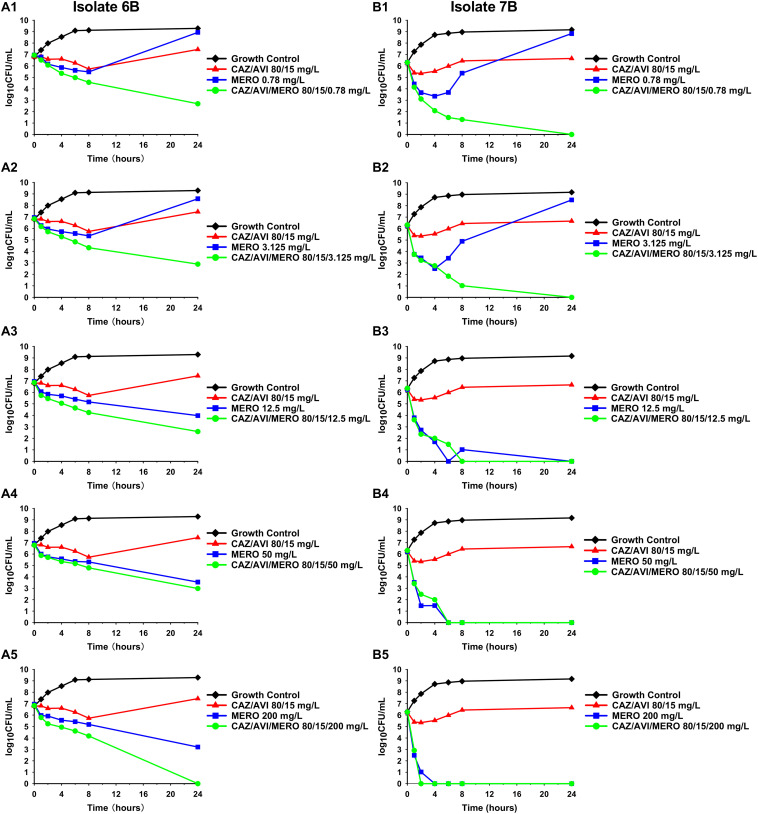
Time-kill assays over 24 h against CAZ/AVI-resistant KPC-Kp isolates. Combinations of CAZ/AVI and MERO were evaluated against 2 CAZ/AVI-resistant KPC-Kp isolates with mutations in the *bla*_KPC_ Ω-loop **(A1–A5,B1–B5)**.

**FIGURE 3:**
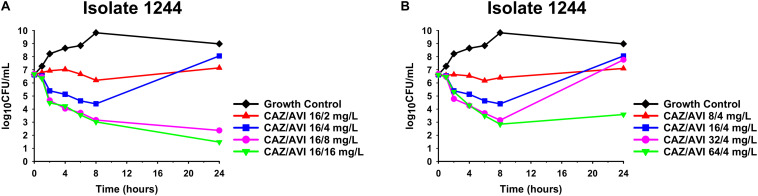
Time-kill assays over 24 h against CAZ/AVI-resistant KPC-Kp isolate 1244. Clinically achievable concentrations of CAZ/AVI were evaluated against CAZ/AVI-resistant KPC-Kp with outer membrane porin mutations and elevated *bla*_KPC_ expression **(A,B)**.

Against CAZ/AVI-susceptible KPC-Kp isolates, CAZ/AVI concentrations ≥ 40/7.5 mg/L were bactericidal at 24 h and caused nearly undetectable viable bacterial counts (5.42 log_10_ CFU/mL mean 24 h killing) (Figures 1A–I). Conversely, CAZ/AVI 20/3.75 mg/L was not bactericidal at 24 h for KPC-Kp 5A, 24A, and 26A and only caused 1.92, 1.27, and 1.21 log_10_ CFU/mL reductions, respectively (Figures 1A,F,H). Furthermore, these three isolates began to regrow in the presence of CAZ/AVI 20/3.75 mg/L between 8 and 24 h. Against CAZ/AVI-resistant KPC-Kp 6B and 7B, bactericidal activity and synergy was observed for the combinations of CAZ/AVI with MERO 0.78 or 3.125 mg/L (Figures 2A1,A2,B1,B2). This combination at the higher MERO concentrations (12.5, 50, and 200 mg/L) displayed moderately enhanced killing compared to monotherapies for KPC-Kp 6B (Figures 2A3–A5). In contrast, MERO at these higher concentrations of ≥12.5 mg/L achieved >6 log_10_ CFU/mL reductions alone against KPC-Kp 7B, thereby negating any potential benefit for the combination (Figures 2B3–B5). Against KP1244, increasing the AVI or the CAZ concentration led to increased and bactericidal activity of CAZ/AVI, even though the isolate was resistant (Figure 3). CAZ/AVI 16/8, 16/16, and 64/4 mg/L resulted in 4.33, 5.20, and 3.04 log_10_ CFU/mL 24 hour reductions against KP1244, respectively, while the lower concentrations regrew to near growth control bacterial density.

## Discussion

Emergence of CAZ/AVI resistance has been observed in 14% of patients with KPC-Kp infections that are treated with CAZ/AVI (Shields et al., 2018a). Thus, there remains a critical need to optimize the use of CAZ/AVI in order to reduce proliferation of resistance and also identify treatment strategies that are active against CAZ/AVI-resistant KPC-Kp. Herein, for 33% (*n* = 3/9) of susceptible isolates, CAZ/AVI 20/3.75 mg/L failed to achieve bactericidal activity and allowed the bacteria to begin regrowing in time-kill assays. The lack of bactericidal activity at ∼20–40× the MIC in some isolates is worrisome, especially since CAZ/AVI concentrations are estimated to be ≤20/3.75 mg/L for ∼20–30% of the dosing interval in patients (Li et al., 2019). Our findings are in agreement with previous time-kill assay results for CAZ/AVI against KPC-Kp that also found regrowth in a subset of the tested isolates at concentrations between 2× and 8× the MIC (Keepers et al., 2014; Shields et al., 2018b). There are a few potential mechanistic explanations for regrowth in the presence of CAZ/AVI. Higher levels of β-lactamase expression in these isolates may be responsible since regrowth only occurred with the lowest AVI concentration in our experiments. Differences in β-lactamase expression were also thought to impair the activity of CAZ/AVI in time-kill assays for a *K. pneumoniae* isolate with *bla*_KPC__–__2_ where increasing AVI concentrations were able to prevent regrowth (Keepers et al., 2014). Amplification of pre-existing CAZ/AVI-resistant subpopulations may also cause regrowth but this was not the cause in the present study as none of the isolates had CAZ/AVI-resistant subpopulations at baseline. It is also possible that these isolates acquired novel stochastic mutations that caused CAZ/AVI resistance, enabling regrowth. Future studies should sequence the isolates after exposure to CAZ/AVI to elucidate the precise mechanism(s) that cause regrowth. These findings highlight the potential importance of sustaining high CAZ/AVI concentrations at the site of infection to suppress the emergence of resistance.

CAZ/AVI resistance in KPC-Kp isolates has developed in an unacceptably high number of patients, yet little data is available to guide antimicrobial therapy for the resistant isolates (Shields et al., 2018a). In the CAZ/AVI-resistant KPC-Kp isolates we generated *in vitro*, KPC-Kp 6B obtained the previously described D179Y substitution in the KPC enzyme’s Ω-loop (Barnes et al., 2017; Shields et al., 2017a) while KPC-Kp 7B developed a novel 21 base pair deletion that was overlapping the Ω-loop encoding portion of the gene. To our knowledge, we are the first report this 21 base pair deletion in a CAZ/AVI-resistant KPC-Kp. Against both of these CAZ/AVI-resistant, MERO-susceptible KPC-Kp isolates, combinations with MERO and CAZ/AVI were synergistic and prevented regrowth at low MERO concentrations. This combination has previously been shown to be synergistic against CAZ/AVI-resistant, MERO-resistant KPC-Kp isolates in E-test synergy assays (Gaibani et al., 2017). We are the first to observe synergy with CAZ/AVI and MERO against CAZ/AVI-resistant and MERO-susceptible KPC-Kp that do not harbor MBL-encoding genes. Regrowth in the presence of MERO alone is in agreement with time-kill assay data for imipenem (4× MIC) against a KPC-Kp with the D179Y substitution (Göttig et al., 2019), where regrowth was observed. However, resistance did not emerge in a separate study with MERO (≥4× MIC) against 4 KPC-Kp isolates with the same D179Y mutation (Shields et al., 2017b). Indeed, KPC-Kp isolates with mutations in the Ω-loop of the *bla*_KPC_ gene have previously been observed to revert to a carbapenem-resistant phenotype *in vitro* (Shields et al., 2017c). Some case reports have been published showing variable success of using carbapenems to treat patients with CAZ/AVI-resistant, MERO-susceptible KPC-Kp (Cano et al., 2019). Unlike MERO monotherapy, the combination of CAZ/AVI and MERO may act synergistically to prevent the reversion to carbapenem resistance. CAZ/AVI and MERO are individually associated with few adverse side effects; in a recent phase three clinical trial that compared CAZ/AVI to MERO for treatment of pneumonia, four patients (1%) that received CAZ/AVI and 0 patients that received MERO were judged by investigators to have serious adverse events due to the study drugs (Torres et al., 2018). However, additional studies are required to confirm that this combination is also well tolerated. Meropenem/vaborbactam or imipenem/relebactam may provide a similar selective pressure and also warrant consideration for KPC-Kp isolates resistant to CAZ/AVI due to mutations in the *bla*_KPC_ gene that lead to carbapenem susceptibility. Against an isolate with increased expression of *bla*_KPC_ in combination with non-functional outer membrane porin channels, we found that increasing the concentration of CAZ or AVI was able to overcome resistance. To our knowledge, this is the first study to show that higher yet clinically achievable concentrations of CAZ/AVI can be bactericidal against resistant isolates. Higher concentrations of the β-lactam and β-lactamase inhibitor may overcome resistance by increasing inhibition of the penicillin binding proteins or the KPC enzyme, respectively. These data may represent a limitation to using a fixed concentration of AVI (4 mg/L) in susceptibility testing since higher AVI concentrations can be achieved in patients. Thus, susceptibilities obtained using fixed concentrations of the β-lactamase inhibitor may not accurately reflect the *in vivo* efficacy of a β-lactam/β-lactamase inhibitor such as CAZ/AVI, especially for isolates with elevated β-lactamase expression (Abodakpi et al., 2019). Although rapid diagnostic tests currently available in the clinic would not detect the mechanism of CAZ/AVI resistance, the phenotypic susceptibility to carbapenems may be a useful surrogate to predict the likely mechanism in KPC-Kp isolates without MBL genes.

There are a few limitations to note in the present study. First, the time-kill assays are performed *in vitro* with fixed antibiotic concentrations and few CAZ/AVI-resistant isolates were investigated. Thus, these experiments may not entirely represent the antibiotics’ efficacy in patients and should be validated in future studies. Second, it is not known if the findings herein will readily translate to KPC-Kp isolates with different sequence types or different KPC variants. However, the isolates in the current study represent those most commonly identified in the United States (van Duin et al., 2020). Third, whole-genome sequencing was not performed on KPC-Kp 6B and 7B so additional mutations outside of those in the *bla*_KPC_ genes cannot be excluded. Though, the results from the MHT experiments support that KPC-Kp 6B and 7B continue to express KPC enzymes but that with greatly reduced carbapenemase activity and increased affinity for CAZ. These data provide further support that substitutions in the KPC enzyme’s Ω-loop caused CAZ/AVI resistance in the isolates from the present study (Barnes et al., 2017).

## Conclusion

In conclusion, our data suggest that the optimal treatment approach for CAZ/AVI-resistant KPC-Kp depends on the mechanism of resistance and that low CAZ/AVI concentrations may permit regrowth of some CAZ/AVI-susceptible KPC-Kp isolates. The combination of CAZ/AVI and MERO is active and can suppress regrowth for CAZ/AVI-resistant, MERO-susceptible KPC-Kp isolates. Clinically achievable concentrations of CAZ/AVI may be sufficient to overcome CAZ/AVI-resistant KPC-Kp isolates that have outer membrane porin mutations and elevated *bla*_KPC_ expression. Additional studies using dynamic *in vitro* pharmacokinetic/pharmacodynamic models and pre-clinical animal models are required to validate the active regimens.

## Data Availability Statement

The raw data supporting the conclusions of this article will be made available by the authors, without undue reservation.

## Author Contributions

YH performed the investigation, curated the data, analyzed the data, and assisted with the initial draft preparation. TW designed the experiments, performed the investigation, analyzed the data, and assisted with the initial draft preparation. OP designed the experiments, analyzed the data, and was involved in the funding acquisition. AR developed the methodology and performed the investigation. LC and BK were responsible for methodology development and experimental investigation. MS was involved in the conceptualization and formal analysis. ZB was responsible for conceptualization, formal analysis, project administration, funding acquisition, and manuscript preparation. All authors contributed to the review and editing of the final article and approved the submitted version.

## Conflict of Interest

The authors declare that the research was conducted in the absence of any commercial or financial relationships that could be construed as a potential conflict of interest.
